# Gaussian Process Regression for Mapping Free EnergyLandscape of Mg^2+^-Cl^−^ Ion Pairing in Aqueous Solution: Molecular Insights and Computational Efficiency

**DOI:** 10.3390/molecules30122595

**Published:** 2025-06-15

**Authors:** Wasut Pornpatcharapong

**Affiliations:** Department of Chemistry, Faculty of Science, Chiang Mai University, Chiang Mai 50200, Thailand; wasut.p@cmu.ac.th

**Keywords:** machine learning, GPR, molecular dynamics, free energy, metadynamics, enhanced sampling, ion pairing, solvation

## Abstract

Free energy landscapes are pivotal for understanding molecular interactions in solution, yet their reconstruction in complex systems remains computationally demanding. In this study, we integrated Gaussian process regression (GPR) with well-tempered metadynamics (WT-MTD) to efficiently map the free energy landscape of the Mg^2^^+^-Cl^−^ ion pairing in an aqueous solution, a system central to biological processes such as magnesium hydration and ligand exchange. We compared traditional umbrella sampling (WHAM) with WT-MTD-derived free energy profiles, identifying critical discrepancies attributed to insufficient sampling in barrier regions. WT-MTD captures two distinct minima corresponding to the contact ion pair (CIP: 0.23 nm) and solvent-separated ion pair (SSIP: 0.47 nm) configurations, consistent with previous computational and experimental studies. GPR, trained on free energy gradients from WT-MTD trajectories, reconstructs smooth landscapes with small datasets (5000 points) while reducing computational costs via grid sparsification. Our results demonstrate that GPR hyperparameters can be optimized based on the insights from WT-MTD simulations, enabling accurate reconstructions even in sparse data regimes. This approach bridges computational efficiency with molecular-level resolution, offering a robust framework for studying ion solvation dynamics and hydration effects in complex systems, where this work is the first application of GPR in ionic solvation environments. The methodology’s scalability to multidimensional landscapes further underscores its potential for advancing molecular simulations in biochemistry and material science.

## 1. Introduction

Free energy simulations have emerged as a critical methodology for identifying metastable states—local free energy minima on the free energy surface—and for elucidating the chemical processes that govern transitions between them. The result of such simulations is a free energy surface, which can be represented as functions of the atomic coordinates within a system [1], where the most probable reaction mechanisms can be inferred from the minimum free energy pathway linking two different minima [2,3,4,5,6,7]. However, expressing free energy landscapes in Cartesian space often complicates the interpretation. To address this, free energy landscapes are typically projected into a collective variable (CV) space, where each CV is defined as a function of collective atomic or residue coordinates that are relevant to the chemical processes or reactions under investigation [8,9].

One of the earliest approaches to computing free energy landscapes is umbrella sampling (US) [1,9]. In this method, the CV space is discretized into uniform grids, with simulations in each grid constrained to sample around a central value, typically using harmonic restraints. The resulting histograms are subsequently analyzed using techniques such as the weighted histogram analysis method (WHAM) or the eigenvector method for umbrella sampling (EMUS) to reconstruct the unrestrained probability distributions [10,11]. These distributions are then used to compute the free energy landscape. However, a significant limitation of US is its computational expense, particularly for high-dimensional CV spaces, as the complexity scales exponentially with the number of CVs (O(*n^D^*), where *D* is the dimensionality of the CV space).

In 2002, Laio and colleagues introduced a novel approach to free energy landscape computation that avoids the need for discretizing the CV space into grids [12]. This method, known as metadynamics (MTD), employs a history-dependent bias potential composed of Gaussian functions, which progressively converges to the true free energy landscape. A refinement of this approach, termed well-tempered metadynamics (WT-MTD), addresses the issue of roughness in the free energy landscape by introducing a tempering parameter (Δ*T* >> *T*) [13,14,15]. In WT-MTD, the height of the deposited Gaussians decreases as the simulation progresses, ensuring that the bias potential converges to a scaled version of the free energy rather than the free energy itself. Other enhanced sampling methods, such as those proposed by Maragliano and Vanden-Eijnden, bias the CVs with higher temperatures, while Darve et al. explored the use of mean force gradients [16,17,18].

Despite the efficiency of MTD and WT-MTD in exploring free energy landscapes, achieving a smooth and accurate representation requires careful parameter selection. For instance, using a small Gaussian height yields a smoother landscape but prolongs simulation times, whereas a larger Gaussian height accelerates exploration at the cost of increased roughness. To balance these trade-offs, recent studies have proposed combining MTD or WT-MTD with machine learning techniques. These hybrid approaches leverage the rapid exploration capabilities of MTD/WT-MTD to generate training data, which is then used to infer the free energy landscape using machine learning algorithms. One such machine learning method is Gaussian process regression (GPR), introduced by Mones et al., which constructs a smooth free energy landscape from trajectories generated by WT-MTD. The training data for GPR consists of free energy derivatives computed at various points along the trajectory [19]. Since WT-MTD trajectories are biased, the influence of the bias potential must be removed to obtain unbiased free energy derivatives, which are then used by GPR to reconstruct the free energy landscape.

In this study, the feasibility of using GPR to reconstruct free energy landscapes was investigated for a real-world chemical system, focusing on the ion pairing process of Mg^2^^+^ and Cl^−^ ions in an aqueous environment, which presents the first application of GPR to reconstruct solvation free energy profiles for ion pairing in an aqueous solution, demonstrating its utility in accurately capturing the thermodynamics of charged species in complex environments. This system is of particular interest due to its relevance to biologically significant reactions, such as ligand exchange around Mg^2^^+^ ions [20,21,22,23,24]. We constructed a one-dimensional free energy landscape based on the Mg^2^^+^-Cl^−^ distance using both US, WT-MTD, and GPR. We evaluated the performance of GPR in terms of computational efficiency, matrix sparsification, and relative errors compared with the reference free energy surface. These analyses were conducted across multiple hyperparameter sets, providing insights into the potential of GPR for reconstructing more complex, multidimensional free energy landscapes in future applications.

## 2. Results and Discussion

### 2.1. Mg^2+^-Cl^−^ One-Dimensional Free Energy Surface

**Figure 1:**
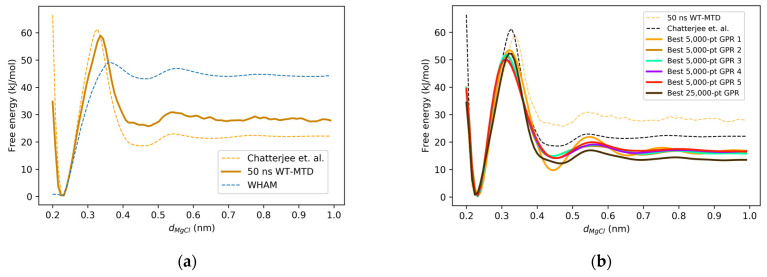
(**a**) Comparison between the free energy landscapes from the WT-MTD simulation, umbrella sampling/WHAM, and the work by Chatterjee et al.; (**b**) comparison between the best GPR reconstruction of the free energy landscapes for each training data, the WT-MTD simulation, and the work by Chatterjee et al.

The one-dimensional free energy landscape, computed using the weighted histogram analysis method (WHAM) with the Mg^2^^+^-Cl^−^ distance (*d*_Mg-Cl_) as the collective variable (CV), exhibits two distinct minima. As illustrated in Figure 1a, the first minimum at *d*_Mg-Cl_ = 0.23 nm corresponds to the contact ion pair (CIP) configuration, while the second minimum at *d*_Mg-Cl_ = 0.47 nm represents the solvent-separated ion pair (SSIP) configuration. The positions of both the CIP and SSIP minima showed strong agreement across our reference simulations (umbrella sampling/WHAM and WT-MTD), as well as with the computational results reported by Chatterjee et al. [25,26,27] and experimental estimates from X-ray and neutron diffraction studies. Furthermore, the free energy barrier location derived from the WT-MTD simulation aligned closely with Chatterjee et al.’s findings, while the WHAM result shows a slight deviation, as summarized in Table 1.

While the locations of the local free energy minima can be easily validated with the experimental results, validating the free energy values is far less trivial. Nevertheless, Figure 1a indicate that for all types of simulations (US/WHAM, WT-MTD, and GPR). The free energy barrier was consistently located at *d*_Mg-Cl_ = 0.34–0.36 nm, which lay approximately midway between the CIP and SSIP minima. These results are consistent with the findings of Chatterjee et al. [27], particularly in terms of the positions of the barrier and the minima. However, discrepancies arise in the absolute values of the free energy barrier and the SSIP-CIP free energy difference. While Chatterjee et al. reported a free energy difference of approximately 20 kJ/mol between the SSIP and CIP basins, our WHAM-derived results indicate a difference of ~45 kJ/mol. Additionally, the free energy barrier relative to the SSIP basin computed using WHAM was less than 10 kJ/mol, compared with the >40 kJ/mol reported by Chatterjee et al. These differences are attributed to insufficient sampling, particularly in the barrier region, as evidenced by the histogram distributions shown in Figure S1 in the Supplemental Materials. Consequently, using the WHAM free energy landscape as a reference for Gaussian process regression (GPR) training may lead to inaccuracies due to these sampling limitations.

### 2.2. GPR Reconstruction of the One-Dimensional Free Energy Surface

To generate training data for GPR, WT-MTD simulations were performed, and the resulting free energy landscapes are presented in Figure 1b. The WT-MTD simulation yielded a free energy profile that aligned more closely with the results of Chatterjee et al. than the WHAM-derived profile. The free energy barriers for the formation of the contact ion pair (CIP) and solvent-separated ion pair (SSIP) were calculated as ~60 kJ/mol and ~30 kJ/mol, respectively, based on the WT-MTD simulation. While these values show qualitative agreement with the general shape of the landscape, some quantitative discrepancies remain, particularly in the relative free energy of the SSIP basin and the bulk region, as illustrated in Figure 1. These deviations are primarily attributed to differences in the force fields used across the simulations. Chatterjee et al. employed the TIP4P water model and OPLS ion potentials, whereas this study used TIP3P water and different ion parameters—a known source of variation in hydration shell energetics. Moreover, as shown in Figure S2 of the Supplementary Materials, the WT-MTD trajectory exhibited superior sampling of rare-event regions compared with umbrella sampling/WHAM, which likely contributed to the observed differences in the barrier heights. Given this relative agreement with the literature, the WT-MTD free energy profile was selected as the reference free energy surface for this study. However, the WT-MTD-derived landscapes exhibited roughness, particularly in the SSIP region and extending into the bulk solvent region, highlighting the need for post-processing to achieve smooth free energy surfaces.

Using multiple 5000-point subsets randomly drawn from the same 25,000-point WT-MTD simulation dataset, GPR successfully reconstructed the 1D free energy landscapes in 7–9 s per hyperparameter set on a PC used in this work. Although GPR hyperparameter selection is often non-trivial, Mones et al. [19] suggested that these parameters can be physically informed by the characteristics of the underlying simulation. In this work, three key hyperparameters were considered: *δ*, function deviation; *σ*, CV uncertainty; and *θ*, the characteristic length scale. Since only one CV was used, both *σ* and *θ* were treated as scalars. The range for *δ* was selected based on the Gaussian height used in the WT-MTD simulation (0.8 kJ/mol) and was swept between 0.4 and 0.9 kJ/mol. The *θ* parameter was informed by the key structural feature of the free energy surface—specifically, the ~0.25 nm separation between the SSIP and CIP basins—and was scanned from 0.20 to 0.35 nm. Although the choice of *σ* was less intuitive, it was empirically swept in the range of 0.05 to 0.15, guided by the scale of noise expected in free energy gradients.

**Figure 2:**
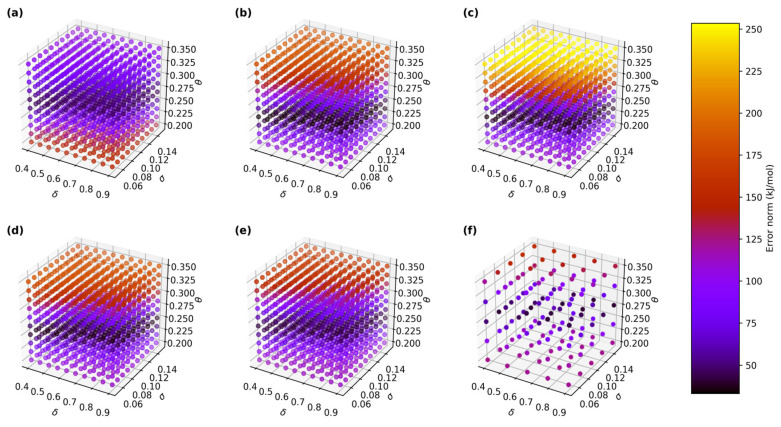
Effects of GPR hyperparameters on error norm of 1D free energy landscape based on *d*_Mg-Cl_ CV relative to WT-MTD reference surface for 5 different randomly sliced 5000-point training data (**a**–**e**) from original 25,000-point training data (**f**).

The relative errors, quantified as the norm of the difference vector between the GPR and the reference free energy surfaces, ranged from 33 kJ/mol to 200 kJ/mol, corresponding to an average error of 0.3–2.0 kJ/mol per data point. The sensitivity of the GPR hyperparameters is summarized in Table 2, which shows that the lowest error norms occurred within a narrow range of *θ* around 0.25 nm, consistent with the physical separation between the SSIP and CIP basins. This supports the hypothesis that optimal *θ* values are closely tied to the underlying system’s geometry. In contrast, the error norms showed little dependence on *δ* (function deviation), indicating that this parameter contributes minimally to reconstruction accuracy. Furthermore, the observed trends remained consistent across all five random 5000-point training datasets, as well as the full 25,000-point dataset, suggesting that the hyperparameter selection protocol is robust and not overly sensitive to data size or sampling variability. Table 2 also summarizes the error norms for various hyperparameter sets, while Figure 2 illustrates the error norms according to various sets of hyperparameters for all five sets of the 5000-point training data and the main 25,000-point training data, where the lowest error norms were concentrated in a similar band of hyperparameters.

When the training data size was increased to 25,000 points, the computational time for GPR increased to 12–15 min per hyperparameter set, reflecting the O(*n*^3^) scaling of the algorithm. Despite the larger dataset, the error norms relative to the reference surface were largely consistent with the smaller 5000-point datasets, with the lowest error norm being 40 kJ/mol. This suggests that increasing the training data size does not necessarily enhance accuracy in this context. The optimum set of hyperparameters for this training data was also in line with that obtained from the 5000-point training datasets (Table 2).

### 2.3. Addressing High GPR Computational Cost in Large Data Regime

To address the computational cost associated with large training datasets, grid sparsification was employed. This approach leverages the sparsity of the Jacobian matrices involved in GPR calculations, which arises from the fact that the *d*_Mg-Cl_ CV involves only two atoms (Mg^2^^+^ and Cl^−^). By assuming sparsity in these matrices, the computational time was reduced by more than an order of magnitude, with each GPR estimation completed in less than 0.2 s.

**Figure 3:**
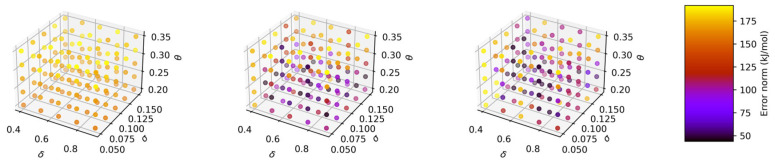
Effects of GPR hyperparameters on error norm of 1D free energy landscape based on *d*_Mg-Cl_ CV relative to WT-MTD reference surface for 25,000-point training data with sparsification grid sizes of 10 (**left**), 25 (**center**), and 50 (**right**).

The grid size for sparsification significantly influenced the error norms. A grid size of 25 yielded the lowest error norm of 40 kJ/mol, comparable to the results obtained with standard GPR. A grid size of 50 produced similar results, while a grid size of 10 resulted in significantly higher error norms, as shown in Table 2 and Figure 3. In terms of the optimum set of hyperparameters, using grid sparsification also yielded similar results to regular GPR. Moreover, sparsification also offered a substantial advantage in terms of computational efficiency, reducing the calculation time by 3–4 orders of magnitude for the 25,000-point training data.

### 2.4. Extending GPR Beyond One-Dimensional Free Energy Reconstruction

While the application of GPR to 1D free energy landscapes may appear to add complexity to WT-MTD simulations, which already provide reasonable estimates of free energy profiles, this work demonstrates that GPR can achieve accurate reconstructions with relatively small training datasets. Furthermore, the result indicates that reasonable estimates for GPR hyperparameters can be derived directly from the underlying enhanced sampling simulation physics. However, determining the globally optimal set may require systematic and computationally intensive parameter sweeps. While this approach demands greater resources, it promises a deeper understanding of how the simulation setup influences GPR performance, especially in higher-dimensional applications. This finding is particularly relevant for multidimensional free energy landscape reconstruction. A potential strategy for extending this approach to multidimensional free energy landscapes is to decompose the problem into multiple one-dimensional subspaces, each corresponding to a single collective variable (CV). As demonstrated in this study, optimal GPR hyperparameters can be identified individually for each CV by comparing 1D GPR reconstructions to their respective reference surfaces. These calibrated hyperparameters can then be used as a foundation for reconstructing high-dimensional free energy surfaces from brief WT-MTD simulations, provided that the sampling sufficiently captures rare-event transitions across the multidimensional CV space.

## 3. Materials and Methods

The system under investigation consisted of 1 Mg^2^^+^ ion, 2 Cl^−^ ions, and 629 TIP3P water molecules enclosed within a cubic simulation box with dimensions of 2.7 × 2.7 × 2.7 nm^3^. All molecular dynamics (MD) simulations were performed using the OpenMM (v8.1.1) software package [28]. The system was initialized, energetically minimized, and equilibrated for 4 ns under the NVT ensemble at a constant temperature of 300 K. Temperature control was achieved using Langevin dynamics with a friction coefficient of 0.5 ps^−1^ and a timestep of 2 fs. The minimization and equilibration steps were executed on GPU hardware accelerated by CUDA (v12). The force field parameters for the Mg^2^^+^ ion were adopted from the work of Schweirz [29], while the parameters for the Cl^−^ ions and TIP3P water molecules were derived from the CHARMM36 force field [30]. Periodic boundary conditions were applied, and long-range electrostatic interactions were treated using the particle mesh Ewald (PME) method, with a cutoff distance of 1.0 nm.

Following equilibration, a one-dimensional (1D) WT-MTD simulation was conducted for 50 ns using the OpenMM-PLUMED (v2.0.1) interface [31]. To investigate the influence of the training data quantity on the Gaussian process regression (GPR) performance, 25,000 data points of free energy derivatives were obtained from the above WT-MTD solution. To assess the robustness of GPR under reduced data conditions, five random subsets of 5000 data points were extracted from this main dataset. The collective variable (CV) for these simulations was defined as the distance between the Mg^2^^+^ ion and one of the Cl^−^ ions (denoted as *d*_Mg-Cl_). The WT-MTD parameters included a biasing factor (*γ*) of 20, an initial Gaussian height of 0.8 kJ/mol, and a Gaussian width of 0.01 nm.

The trajectories generated from the WT-MTD simulations were processed using the mdTraj (v1.10.0) library [32]. The biased free energy derivatives and WT-MTD bias potentials were computed following the methodology outlined by Mones et al. In this work, the Jacobian matrices of the CV with respect to atomic coordinates were calculated analytically, rather than using numerical approximations. Unbiased free energy derivatives were obtained by subtracting the WT-MTD bias contributions from the biased derivatives. These unbiased derivatives served as the input for GPR, which was employed to reconstruct the free energy landscape using the software provided by Mones et al. [19], available via the GitHub repository cited in their publication through the following link: https://github.com/molet/program_gp_general (accessed on 20 May 2025). All simulation packages, including OpenMM, OpenMM-PLUMED, and mdTraj, were installed using Anaconda on a local workstation running Python 3.12.5. The processing of both biased and unbiased free energy derivatives was performed using custom Python scripts developed by the author, strictly following the implementation guidelines outlined in Mones et al.

To validate the free energy landscapes reconstructed via GPR, a 1D umbrella sampling (US) simulation was performed along the *d*_Mg-Cl_ coordinate. The US simulation spanned 49 windows, covering a *d*_Mg-Cl_ range from 0.206 to 0.99 nm. The initial configurations for each window were generated using steered molecular dynamics (SMD), during which the *d*_Mg-Cl_ CV was pulled at a rate of 0.004 nm per step over 2,000,000 steps. Each window was subsequently simulated for 4 ns under a harmonic restraint with a spring constant of 5000 kJ/mol/nm^2^. Histogram data were recorded every 100 steps during the umbrella sampling simulations, and the resulting distributions were unbiased using the weighted histogram analysis method (WHAM) to construct a reference one-dimensional free energy profile using the software provided by Grossfield (v2.1.0) [10]. This WHAM-derived profile was then compared with both the WT-MTD results and those reported in the literature [27]. The surface that most closely matched the literature data was selected as the reference benchmark for evaluating the accuracy of the GPR-reconstructed free energy landscapes.

The GPR-reconstructed free energy landscapes were optimized by minimizing the norm of the difference between the GPR-derived surfaces and the reference free energy profile. The GPR hyperparameters—function deviation (*δ*), CV deviation (*σ*), and length scale (*θ*)—were systematically varied within the ranges of 0.40–0.90, 0.05–0.15, and 0.20–0.35, respectively. Grid sparsification of the GPR matrices was also implemented with grid sizes of 10, 25, and 50 to assess the computational efficiency when applying them to the 25,000-point dataset. The relative errors between the GPR and the reference free energy surfaces were quantified by computing the norm of the difference vector between the two surfaces at 100 data points. Additionally, computational performance metrics, including the time required for GPR estimation, were obtained from simulations on a desktop personal computer (PC) with an 8-core AMD Ryzen^TM^ 7 5800X CPU, with 32GB of DDR4 RAM and Nvidia GeForce^TM^ RTX 2060 GPU for CUDA acceleration of MD simulations, with NVMe storage for fast data I/O. All the GPR performance benchmarks, according to the aforementioned specifications, were recorded for both training datasets.

## 4. Conclusions

In this study, the feasibility of combining well-tempered metadynamics (WT-MTD) with Gaussian process regression (GPR) to reconstruct free energy landscapes, using the ion-pairing process of Mg^2^^+^ and Cl^−^ in water as a test case, was thoroughly investigated. The results demonstrate that WT-MTD provides a robust framework for exploring CV spaces, yielding free energy profiles that align well with the literature, albeit with some roughness. GPR, trained on free energy derivatives from WT-MTD trajectories, effectively smooths and reconstructs the free energy landscape, with relatively small training datasets. Grid sparsification further enhances the computational efficiency in a large training data regime, reducing calculation times by several orders of magnitude without a significant loss of accuracy.

The discrepancies observed between the WHAM-derived free energy profiles and literature values underscore the importance of adequate sampling, particularly in barrier regions. WT-MTD, combined with GPR, addresses this challenge by enabling the rapid exploration of CV spaces and providing a data-driven approach to free energy reconstruction. This approach is particularly promising for multidimensional systems, where the scalability of traditional methods is limited. By leveraging the sparsity of Jacobian matrices and optimizing hyperparameters based on insights from WT-MTD simulations, GPR offers a pathway to reconstructing complex free energy landscapes with minimal computational overhead.

Future work will focus on extending this methodology to higher-dimensional CV spaces and more complex systems, such as biomolecular processes, where efficient and accurate free energy calculations are critical. The integration of machine learning techniques like GPR with enhanced sampling methods represents a powerful tool for advancing our understanding of chemical and biological systems.

## Figures and Tables

**Table 1:** Comparison of key features in the free energy landscapes obtained from umbrella sampling/WHAM and WT-MTD simulations in this work, alongside literature values from Chatterjee et al. and experimental estimates from X-ray and neutron diffraction studies. The table lists the positions (in nm) of the contact ion pair (CIP), solvent-separated ion pair (SSIP), and the free energy barrier. Experimental values are not direct Mg^2^^+^-Cl^−^ distances but reflect the approximate locations of the first and second hydration shells. These comparisons support the accuracy of the simulated CIP and SSIP configurations and highlight the importance of enhanced sampling methods like WT-MTD in capturing barrier regions consistently with experimental observations.

	WHAM	WT-MTD	Chatterjee et al.	Experimental
CIP Location (*d*_Mg-Cl_ nm)	0.23	0.23	0.23	0.20–0.22
SSIP Location (*d*_Mg-Cl_ nm)	0.47	0.47	0.47	0.41–0.43
Barrier Location (*d*_Mg-Cl_ nm)	0.36	0.34	0.33	n/a

**Table 2:** Various sets of GPR hyperparameters corresponding to lowest error norms relative to reference surface in their respective training models, with errors per data point for each set of training criteria shown.

Training Data/GPR Models	*δ*	*σ*	*θ*	Error Norm (kJ/mol)	Error per Data Point (kJ/mol)
5000 points/regular (Batch 1)	0.40	0.11	0.25	44.35	0.44
5000 points/regular (Batch 1)	0.57	0.15	0.25	44.35	0.44
5000 points/regular (Batch 2)	0.40	0.14	0.22	33.34	0.33
5000 points/regular (Batch 2)	0.62	0.13	0.23	33.82	0.34
5000 points/regular (Batch 2)	0.73	0.15	0.23	33.80	0.34
5000 points/regular (Batch 3)	0.40	0.14	0.22	35.09	0.35
5000 points/regular (Batch 3)	0.62	0.13	0.23	35.40	0.35
5000 points/regular (Batch 3)	0.79	0.15	0.23	35.24	0.35
5000 points/regular (Batch 4)	0.46	0.12	0.23	36.17	0.36
5000 points/regular (Batch 4)	0.51	0.13	0.23	36.03	0.36
5000 points/regular (Batch 4)	0.84	0.13	0.25	36.68	0.37
5000 points/regular (Batch 5)	0.40	0.13	0.23	39.90	0.40
5000 points/regular (Batch 5)	0.51	0.15	0.23	40.54	0.41
5000 points/regular (Batch 5)	0.79	0.15	0.25	40.65	0.41
25,000 points/regular	0.40	0.08	0.28	40.15	0.40
25,000 points/regular	0.65	0.13	0.28	40.34	0.40
25,000 points/regular	0.90	0.15	0.28	40.73	0.41
25,000 points/sparse grid size = 10	0.40	0.15	0.20	158.13	1.58
25,000 points/sparse grid size = 25	0.40	0.10	0.24	45.36	0.45
25,000 points/sparse grid size = 25	0.53	0.15	0.24	45.13	0.45
25,000 points/sparse grid size = 25	0.90	0.05	0.24	46.85	0.47
25,000 points/sparse grid size = 50	0.40	0.13	0.24	43.85	0.44
25,000 points/sparse grid size = 50	0.53	0.10	0.28	47.50	0.48
25,000 points/sparse grid size = 50	0.78	0.15	0.24	47.20	0.47

## Data Availability

The data presented in this study are available on request from the corresponding author.

## Supplementary Materials

The following supporting information can be downloaded at https://www.mdpi.com/article/10.3390/molecules30122595/s1. Figure S1: Histogram of *d*_Mg-Cl_ CV collected from umbrella sampling (US) simulations; Figure S2: *d*_Mg-Cl_ CV sampling histograms from the 50 ns WT-MTD simulation for all 5 randomly sliced 5000-point datasets and the main 25,000-point dataset.
