# Supplementary material for: Gaussian Process Regression for Mapping Free EnergyLandscape of Mg2+-Cl− Ion Pairing in Aqueous Solution: Molecular Insights and Computational Efficiency
Source: Molecules. 2025 Jun 15;30(12):2595. doi: 10.3390/molecules30122595 (PMC12196332; doi:10.3390/molecules30122595)
Supplement: Supplementary file 1 [file molecules-30-02595-s001.zip › molecules-3683976-supplementary.pdf]

Gaussian Process Regression for Mapping Free Energy Landscape of Mg^{2+}-Cl^{-} Ion Pairing in Aqueous Solution: Molecular Insights and Computational Efficiency

Wasut Pornpatcharapong

Department of Chemistry, Faculty of Science, Chiang Mai University, Chiang Mai 50200, Thailand; wasut.p@cmu.ac.th

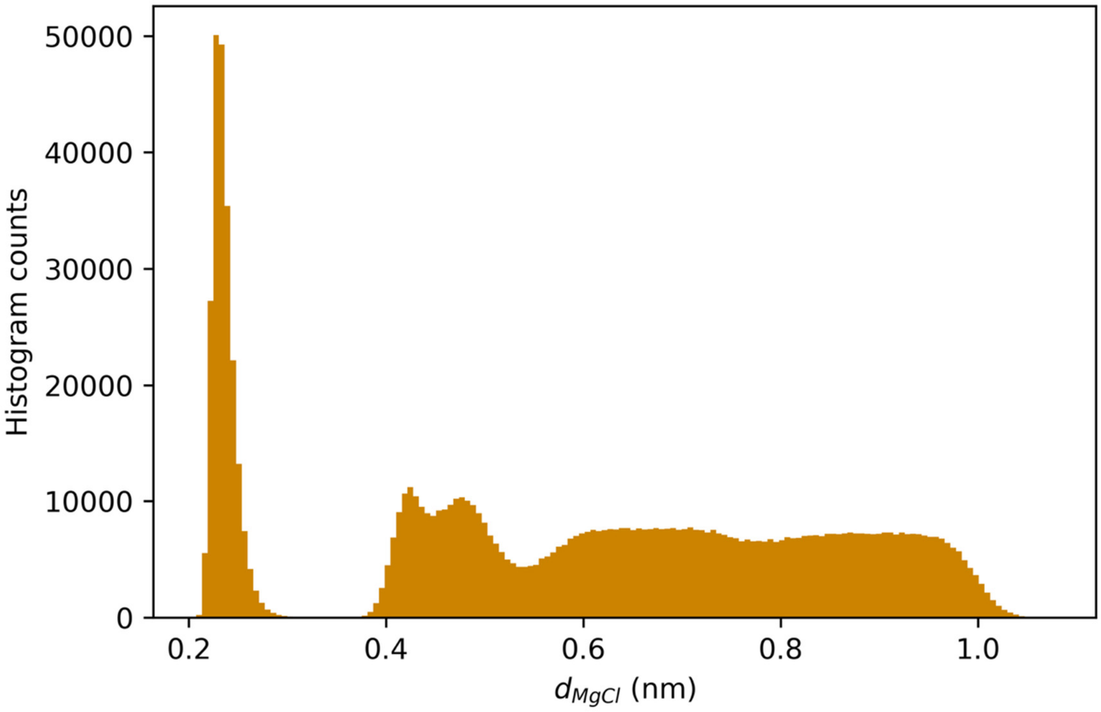

Figure S1: Histogram of $d_{\text{Mg-Cl}}$ CV collected from umbrella sampling (US) simulations.

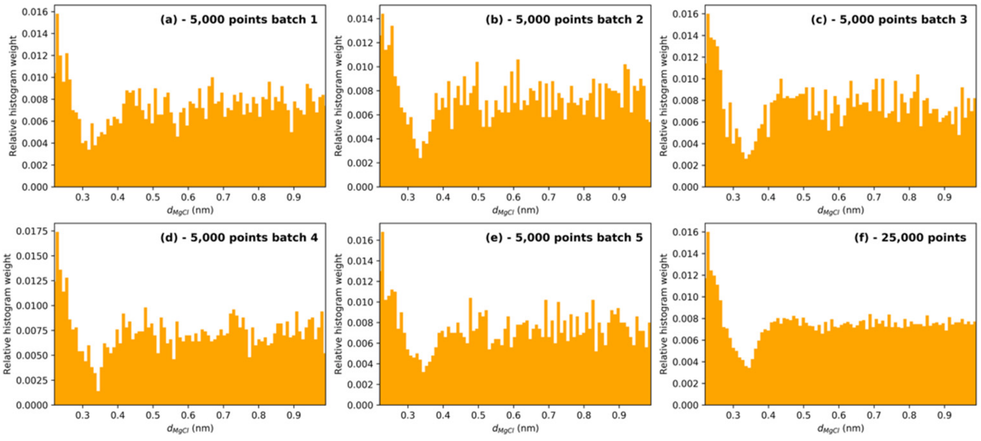

Figure S2: $d_{\text{Mg-Cl}}$ CV sampling histograms from the 50 ns WT-MTD simulation for all 5 randomly sliced 5,000-point datasets and the main 25,000-point dataset.
